# Extracellular vesicle-associated lncRNA LYPLAL1-DT mediates endothelial-cancer cell communication, promoting small cell lung cancer progression

**DOI:** 10.20517/evcna.2025.119

**Published:** 2025-12-19

**Authors:** Xing Zhang, Caijiao Hu, Zhihui Li, Jing Lu, Xueyun Huo, Lixue Cao, Meng Guo, Changlong Li, Xin Liu, Zhenwen Chen, Jianyi Lv, Xiaoyan Du

**Affiliations:** ^1^School of Basic Medical Sciences, Capital Medical University, Beijing 100069, China.; ^2^Laboratory for Clinical Medicine, Capital Medical University, Beijing 100069, China.; ^3^Beijing Key Laboratory of Cancer Invasion and Metastasis Research, Beijing 100069, China.; ^#^These authors contributed equally to this work.; ^†^The author served as senior authors.

**Keywords:** LYPLAL1-DT, exosome, endothelial cells, PFN2, BCL2, SIRT1, SCLC

## Abstract

**Aim:** Representing about 15% of lung cancers, small cell lung cancer (SCLC) is an extremely aggressive disease characterized by rapid growth and early spread, leading to dismal clinical outcomes. In this study, we aimed to investigate the dual roles of exosomal long non-coding RNA (lncRNA) LYPLAL1-DT (LYPLAL1 divergent transcript) in both tumor cells and vascular endothelial cells.

**Methods:** The circulating levels of LYPLAL1-DT were measured using real-time polymerase chain reaction in 13 SCLC patients and 21 normal controls. Exosomes from the supernatant of cell culture medium or serum were extracted through ultracentrifugation and dyed with PKH67 green fluorescent cell linker to identify internalization. Migration and invasion assay, colony formation, Cell Counting Kit-8 (CCK-8), and tube formation assays were used to assess the malignant effects of extracellular RNAs (exRNAs) LYPLAL1-DT in exosomes.

**Results:** Exosomal LYPLAL1-DT is upregulated in SCLC patients and plays a dual role in promoting tumor cell aggressiveness and enhancing pro-angiogenic behavior in endothelial cells, thereby accelerating SCLC progression. Mechanistically, LYPLAL1-DT functions as a competing endogenous RNA, exerting its effects through the miR-204-5p/profilin-2, miR-204-5p/B-cell lymphoma 2 and miR-204-5p/sirtuin 1 regulatory axes. These pathways underscore the pleiotropic effects of exosomal LYPLAL1-DT and underscore its value as a promising therapeutic target.

**Conclusion**: In the current study, we investigated the bidirectional communication mediated by exRNA LYPLAL1-DT between SCLC and endothelial cells, while also exploring its potential regulatory targets. This research provides a potential circulating biomarker for the diagnosis, prognosis, and treatment of SCLC.

## INTRODUCTION

Small cell lung cancer (SCLC) represents a highly malignant form of lung cancer, comprising about 15% of cases. It is marked by rapid proliferation, early dissemination, and the development of therapy resistance, all contributing to a dismal prognosis^[1]^. Despite advances in immunotherapy and targeted therapies, the five-year survival rate of SCLC remains below 5%. Therefore, understanding the pathological mechanisms and regulatory networks in SCLC is crucial for identifying novel therapeutic targets^[2]^.

Extracellular RNAs (exRNAs), a heterogeneous population of different ribonucleic acids that are found in all biofluids, play vital roles in various physiological and pathological processes^[3]^. In tumor progression, they have emerged as critical mediators of intercellular communication within the tumor microenvironment (TME). Released by tumor cells and surrounding stromal cells, exRNAs are often encapsulated in extracellular vesicles (EVs) such as exosomes, enhancing their stability in the extracellular space. These molecules can regulate key processes, including angiogenesis, immune evasion, metastasis, and chemoresistance^[4]^. Recent studies have gradually deciphered the key roles of exRNAs, especially microRNAs (miRNAs) and long non-coding RNAs (lncRNAs), in tumor development and progression^[5,6]^. It has been reported that lncRNA HOX transcript antisense RNA (HOTAIR) promotes exosome secretion of hepatocellular cancer cells by regulating Ras-Related Protein Rab-35 (Rab35) and Synaptosome Associated Protein 23 (SNAP23)^[7]^. Long intergenic non-protein coding RNA 461 (LINC00461) from mesenchymal stem cell-derived exosomes promoted multiple myeloma tumorigenesis by targeting miR-15a/16/B-cell lymphoma 2 (BCL2) axis^[8]^. In hypoxic conditions, the lncRNA urothelial carcinoma-associated 1 (UCA1) was upregulated in cancer cell-derived exosomes and contributed to cancer progression via multiple signaling pathways^[9]^. These findings highlight the vital roles of exRNAs in mediating crosstalk between tumor cells and the TME.

As an important component of the TME, endothelial cells critically drive tumor progression by orchestrating angiogenesis and shaping the TME^[10]^. AI-Nedawi *et al*. demonstrated that the transfer of the oncogenic constitutively active epidermal growth factor receptor variant III (EGFRvIII) via EVs can transmit oncogenic activity among cancer cells and active vascular endothelial growth factor (VEGF) signaling in endothelial cells^[11]^. Enriched with hypoxia-induced RNAs and proteins, tumor-derived extracellular EVs play a key role in regulating endothelial cell function and enhancing vascular permeability^[12,13]^. In our previous study, we identified lncRNA LYPLAL1-DT (LYPLAL1 divergent transcript) significantly enhances the proliferation and migration of endothelial cells under inflammatory and high glucose conditions to protect against type 2 diabetes with macrovascular complications^[14]^. More recently, we found that LYPLAL1-DT was upregulated by the RNA-binding protein ELAV-like RNA binding protein 4 (ELAVL4), and positively correlated with the malignant phenotype of SCLC^[15,16]^. Several key pathways are implicated in SCLC progression and tumor-endothelial interactions, most notably notch receptor (NOTCH), mechanistic target of rapamycin kinase (mTOR) and mitogen-activated protein kinase (MAPK) signaling pathways^[2,17]^. Our team has demonstrated that LYPLAL1-DT functions as a competing endogenous RNA (ceRNA) by downregulating miR-204-5p, thereby promoting expression of its downstream targets profilin-2 (PFN2), BCL2 and sirtuin 1 (SIRT1)^[14-16]^. PFN2, a small actin-binding protein, has been shown to modulate cytoskeletal dynamics, cellular migration and angiogenesis of cancer cells^[15,18-20]^. BCL2 acts as a pivotal regulator that orchestrates the interplay between apoptosis and autophagy, involved in multiple processes of tumor cells^[21,22]^. SIRT1, a NAD^+^-dependent deacetylase, influences tumor cell metabolism, apoptosis, and angiogenesis^[23,24]^. This delineates the regulatory network of LYPLAL1-DT in the development of SCLC. However, its roles in remodeling the TME remain elusive.

In the present study, we clarified the bidirectional crosstalk mediated by exRNA LYPLAL1-DT between SCLC and endothelial cells, while also conducting preliminary explorations into its potential regulatory targets. This research offers a dynamic biomarker for the diagnosis, prognosis, and treatment of SCLC.

## METHODS

### Serum samples

We collected serum samples from 13 SCLC patients and 21 normal controls at Beijing Chest Hospital (China) between March 2024 and December 2024. Ethical approval was obtained from the institutional review board. All procedures involved in sample collection were performed with written informed consent from the patients or their guardians.

### Cell culture

Human SCLC cell lines (NCI-H446, NCI-H196, DMS114) and the umbilical vein endothelial cell line (HUVEC) were obtained from the National Cell Line Resource Platform, China. Cell lines were cultured in RPMI 1640 medium supplemented with 10% fetal bovine serum (FBS, VisTech, New Zealand). All cell lines were cultured at 37 °C in 5% CO_2_.

### Exosomes isolation and identification

Exosome-free FBS was prepared via ultracentrifugation (100,000 × *g*, 4 °C, overnight) to deplete endogenous bovine exosomes, followed by sterile filtration (0.22 μm, Millipore)^[25,26]^. H446 and HUVEC cells were then cultured in medium containing this exosome-depleted FBS for 48 h. Once the cells reached approximately 90% confluence, the culture supernatant was collected and sequentially centrifuged first at 3,000 × *g* for 10 min and then at 10,000 × *g* for 30 min (both at 4 °C) to eliminate cellular debris and larger vesicles. The clarified supernatant was subsequently ultracentrifuged at 100,000 × *g* for 90 min at 4 °C. The final pellet containing exosomes was resuspended in 50-100 μL of PBS.

Serum exosomes from patients with SCLC and controls were isolated using the VEX exosome isolation reagent (Vazyme, China, Cat# R602) according to the manufacturer’s instructions. Following isolation, the exosomes were characterized using transmission electron microscopy (TEM) and nanoparticle tracking analysis (NTA). The purified exosome samples were either used immediately for subsequent analysis or stored at -80 °C. The exosomal protein concentration was quantified with a bicinchoninic acid (BCA) protein assay kit (NCM Biotech, USA, Cat# WB6501).

### Lentivirus packaging and construction of stable transfected cell lines

The establishment of LYPLAL1-DT-overexpressing H446 and DMS114 cells, and LYPLAL1-DT knockdown H196 cells, was described previously^[15,16]^. The short hairpin RNAs (shRNAs) targeting the LYPLAL1-DT transcript were listed in Table 1.

**Table 1:** Sequences of shRNAs targeting LYPLAL1-DT transcript

**Name**	**Sequences (5’-3’)**
shRNA-1-top strand	GATCCGGGTCTTATACTGGCTACCTCCTATTTCAAGAGAA TAGGAGGTAGCCAGTATAAGACCCTTTTTTG
shRNA-1-bottom strand	ATTCAAAAAAGGGTCTTATACTGGCTACCTCCTATTCTCTT GAAATAGGAGGTAGCCAGTATAAGACCCG
shRNA-2-top strand	GATCCGCAGATGGAGTTTAGGAGCAAAGTTATTCAAGAGA TAACTTTGCTCCTAAACTCCATCTGTTTTTTG
shRNA-2-bottom strand	AATTCAAAAAACAGATGGAGTTTAGGAGCAAAGTTATCTC TTGAATAACTTTGCTCCTAAACTCCATCTGCG
shRNA-3-top strand	GATCCGCATCTGATGTGATTTCTCAGCTAATTTCAAGAGAAT TAGCTGAGAAATCACATCAGATGTTTTTTG
shRNA-3-bottom strand	AATTCAAAAAACATCTGATGTGATTTCTCAGCTAATTCTCTT GAAATTAGCTGAGAAATCACATCAGATGCG

shRNA: Short hairpin RNA; LYPLAL1 divergent transcript.

The knockdown efficiency of LYPLAL1-DT has been validated as we previously described^[16]^.

### Exosome internalization and exosome-co-culture assay

For the exosome-internalization experiment, 1 μg PKH67 dye (green fluorescence, Biolaibo, Cat# HR8659) and the extracted exosome suspension were used according to the instructions. The staining reaction was terminated with an equal volume of 1% bovine serum albumin in PBS. Labeled exosomes were then ultracentrifuged (100,000 × *g*, 70 min) to remove unincorporated dye and resuspended in PBS. Recipient cells were incubated with the PKH67-labeled exosomes for 6 h, followed by three washes with PBS. Thereafter, the cells were fixed in 4% paraformaldehyde for 10 min and rinsed again with PBS. The cells were then treated with Hoechst for 5 min, washed again and added to the anti-quench blocking solution. The images were observed under a Leica TCS SP5 confocal microscope with a 63× objective.

For the exosome co-culture assay, 6 × 10^4^ cells per well were seeded in 6-well plates, and after a certain period of culture, the medium was replaced with complete medium containing 5 μg/mL exosomes that had been filtered through a sterile cell filter with 0.22 μm pore size to remove residual cell debris. H446 or HUVEC cells were treated with complete medium for 24 h following the protocol described in the references^[27,28]^.

The uptake was quantified through fluorescence intensity analysis using ImageJ software on confocal microscopy images (Leica TCS SP5). Briefly: (1) PKH67-labeled exosomes were incubated with cells; (2) after washing, z-stack images were acquired under standardized exposure; (3) mean fluorescence intensity per cell was measured after background subtraction. The data represent three independent biological replicates (each with technical triplicates), with ten randomly selected fields of view (FOVs) analyzed.

### Flow cytometry analysis

To detect exosome uptake, exosomes were labeled with 1 × PKH67 dye working solution (Beyotime, China, Cat#C3635S). The labeling reaction was terminated by adding the fluorescent labeling stop solution. H446 or HUVEC cells were seeded in 6-well plates at a density of 1 × 10^5^ cells per well. Once the cells reached adequate confluence, the medium was changed for sterile-filtered (0.22 μm) complete medium containing 10 μg/mL exosomes. Cells were subsequently incubated at 37 °C with 5% CO_2_ for 6 h. After incubation, cells were digested with trypsin, collected, and washed twice with PBS. The cell pellet was resuspended in 500 μL PBS and subjected to flow cytometric analysis using a BD Fortessa instrument (Becton, Dickinson and Company, USA) to measure PKH67 fluorescence intensity. Cells incubated with unlabeled exosomes served as the negative control to establish background signal levels.

### Western blot analysis and Quantitative RT-PCR analysis

Western blot and quantitative real-time polymerase chain reaction (qRT-PCR) analyses were performed as previously reported^[1,2]^.

The following antibodies were used in this study: anti-PFN2 (Abcam, Cat#ab191054), anti-BCL2 (Abways, CY6717), anti-β-actin (HUABIO, Cat#R1207-1), anti-SIRT1 (Abcam, Cat#ab287914), anti-CD63 (Abcam, Cat#ab59479), anti-CD81 (Abcam, Cat#ab109201), anti-Alix (Proteintech, Cat#12422-1-AP), and anti-Calnexin (HUABIO, Cat#ER1803-42). A horseradish peroxidase (HRP)-conjugated anti-rabbit IgG secondary antibody (Cell Signaling Technology, Cat# 7074S), diluted 1:10,000 in Tris-buffered saline with Tween 20 (TBST) buffer supplemented with 0.5% non-fat dry milk. The membranes were incubated with the secondary antibody for 1 h at room temperature, followed by enhanced chemiluminescence (ECL) detection.

### Cell co-culture assay

LYPLAL1-DT-overexpressing HUVECs (HUVEC-OE) or control HUVECs (HUVEC-OC) (1.5 × 10^5^ cells/well) were seeded in the upper chamber inserts (0.4 μm pore membrane; Merck, Cat#PICM01250), while wild-type H446 cells (3 × 10^5^ cells/well) were placed in the lower chamber. Both cell types were maintained in RPMI 1640 medium supplemented with 10% exosome-free FBS throughout the 24-h co-culture period.

### Cell proliferation

The cell proliferation rate was assessed using CCK-8 staining. The number of cells was adjusted to 3,000/100 μL using a cell counting plate and 1,640 complete medium, and 100 μL of seed cell suspension was spread in a 96-well plate; that is, 3,000 cells were added to each well. After 4 h of incubation, 10 μL of CCK-8 reagent (Vazyme, Cat#A311-02) was added to each well at a volume of 10 μL. The plates were subsequently incubated for an additional 2 h at 37 °C before the absorbance was measured at 450 nm.

### Transwell migration and invasion assays

Cell migration and invasion were assessed using Transwell chambers (Corning, USA, Cat#3422) and Matrigel-coated Transwell inserts (Corning, Cat#354480), respectively. In both assays, 2 × 10^4^ cells suspended in serum-free RPMI 1640 medium were seeded into the upper chamber, while the lower chamber was filled with 600 μL of complete medium. After 24 h of incubation at 37 °C, cells that had traversed the membrane were fixed and stained with 0.1% crystal violet. The membranes were then washed with PBS, and cells on the lower surface were imaged and quantified under a microscope. All experiments were performed in triplicate.

### Tube formation assay

HUVEC cells were diluted in serum-free RPMI 1640 and added at 1 × 10^4^/well to 96-well culture plates precoated with basement membrane matrix (Corning). The plates were incubated for 12 h at 37 °C. The tubular structures were observed under an inverted microscope, and random selections were made from three different FOVs.

For tube formation analysis, binary-converted (Otsu thresholding) and skeletonized images were processed using the AnalyzeSkeleton plugin to quantify total tube length and branch points as described in the references^[29-31]^.

### Plate colony formation

Cells of each group were seeded with 200 cells per dish in 10 mL medium. After 2-3 weeks of culture, the cells were washed with PBS and then fixed with 4% cell tissue fixator (APPLYGEN, Cat#B1057) for 10 min before staining with 1% crystal violet solution (APPLYGEN, Cat#B1087). Finally, a representative image from random sampling was selected under the microscope for the photograph.

### Statistical analysis

Statistical analyses were conducted using GraphPad Prism 8. Data are expressed as mean ± standard deviation (SD). Differences between groups were assessed by Student’s *t*-test or one-way analysis of variance (ANOVA), as appropriate. *P*-value < 0.05 was considered statistically significant. Flow cytometry data were analyzed using FlowJo 10.8.1. Graphical abstracts and schematic diagrams were generated using Adobe Illustrator 2022.

## RESULTS

### Exosomal LYPLAL1-DT is upregulated in SCLC patients and internalized by SCLC cells

**Figure 1:**
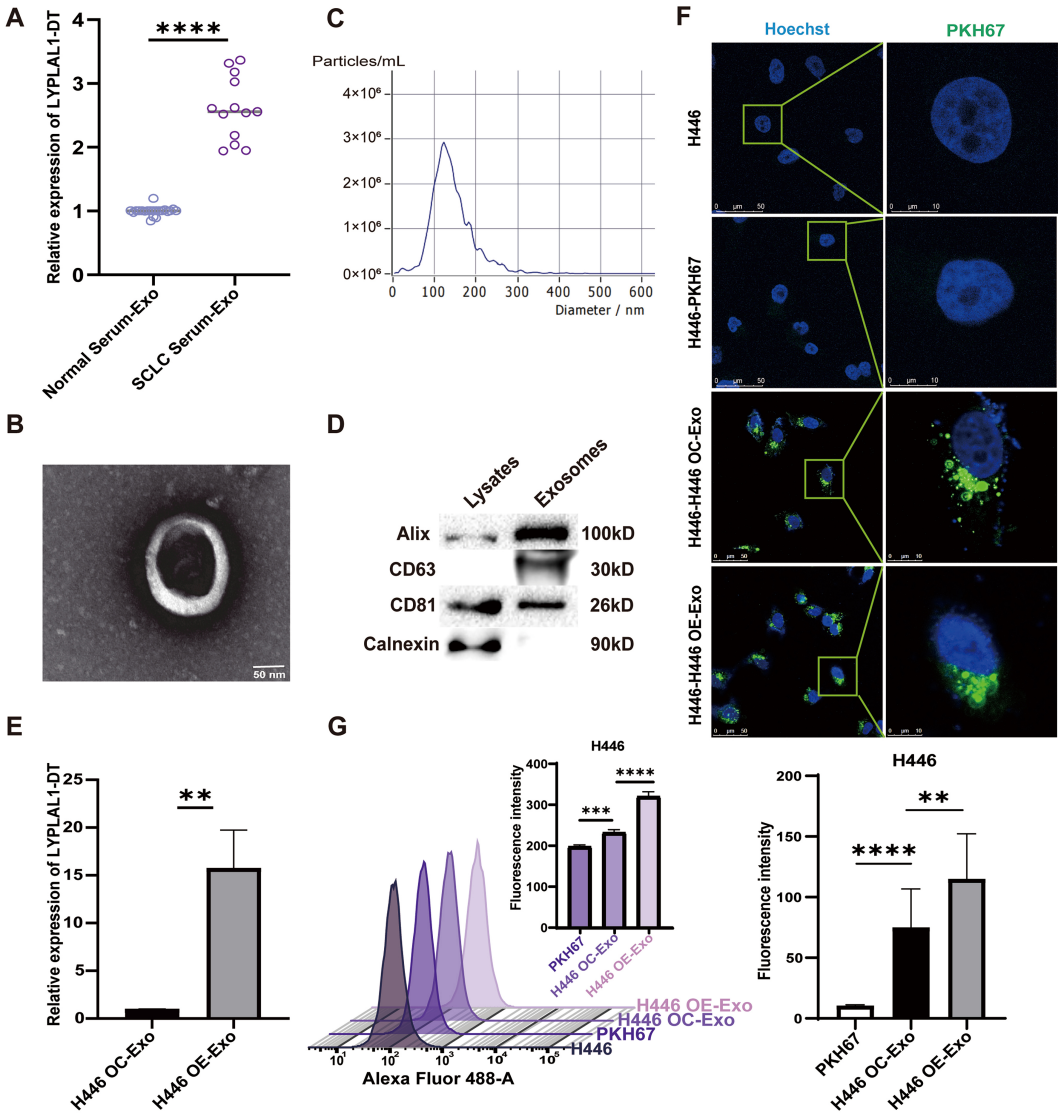
The exRNA LYPLAL1-DT is upregulated in SCLC patients and internalized by SCLC cells. (A) The expression level of LYPLAL1-DT in serum exosomes of SCLC patients and normal controls; (B) Exosomes were confirmed by electron microscopy (scale bar: 50 nm); (C) NTA analysis of exosomes; (D) Detection of exosome markers by Western blotting; (E) Detection of LYPLAL1-DT in exosomes secreted by LYPLAL1-DT–OE and OC H446 cells; (F) Internalization of PKH67-labeled exosomes assessed by fluorescence microscopy (scale bar: 50 and 10 μm); (G) Internalization of PKH67-labeled exosomes assessed by flow cytometry. Data are presented as mean ± SD. Differences between the two groups were analyzed by Student’s *t*-test, whereas comparisons among more than two groups were analyzed by one-way ANOVA. (^*^P < 0.05; ^**^P < 0.01; ^***^P < 0.001; ^****^P < 0.0001). exRNA: Extracellular RNA; LYPLAL1-DT: LYPLAL1 divergent transcript; SCLC: small cell lung cancer; NTA: nanoparticle tracking analysis; OE: overexpression; OC: overexpression control; SD: standard deviation; ANOVA: analysis of variance; PKH67: PKH67 green fluorescent cell linker.

In our previous study, we determined the circulating levels of LYPLAL1-DT in 46 SCLC patients and 18 normal controls. A significantly higher circulating level of LYPLAL1-DT was observed in the SCLC cohort compared to the controls^[15]^. Given the abundance of RNases in extracellular environments, growing evidence demonstrates that circulating exRNAs in blood are protected within EVs upon release^[32,33]^. Thus, we deduced that LYPLAL1-DT was packaged in exosomes and entered the bloodstream from SCLC cells. To verify this hypothesis, we extracted exosomes from the serum of 13 SCLC patients and 21 normal controls. It indicated that the level of LYPLAL1-DT in the exosome of SCLC group was significantly higher than that in the normal group [Figure 1A].

We extracted exosomes secreted by LYPLAL1-DT-overexpressed H446 cells (H446-OE) and control cells (H446-OC)^[15]^, which are defined as H446 OE-Exo and H446 OC-Exo. According to the Minimal Information for Studies of Extracellular Vesicles 2023 (MISEV2023) guidelines, the structure, particle size, concentration, and protein composition analysis were measured individually. Under electron microscopy, the exosomes exhibited a typical cupped structure [Figure 1B]. Their diameter ranged from 100 to 200 nm, with a main concentration around 120 nm, and the particle concentration was close to 3 × 10^6^/mL [Figure 1C]. The markers for exosome identification (Alix, CD63, CD81 and Calnexin) were detected by Western blotting [Figure 1D]. The LYPLAL1-DT level in H446 OE-Exo was significantly higher than in H446 OC-Exo, as determined by qRT-PCR detection [Figure 1E]. These results suggest that LYPLAL1-DT is encapsulated in exosomes secreted by SCLC cells, and its exosomal content is positively correlated with its expression level. Studies have shown that exosomes can mediate intercellular communication in different ways^[34,35]^. These vesicles can fuse with or be endocytosed by recipient cells, thereby delivering their molecular cargo to modulate cellular functions^[36]^. We performed the uptake assay to investigate whether the extracted exosomes could be successfully taken up by SCLC cells. PKH67 labeling revealed green fluorescence in H446 cells [Figure 1F], while flow cytometry detected a clear rightward peak shift after the addition of exosomes [Figure 1G]. These confirmed that both H446 OE-Exo and control H446 OC-Exo were effectively internalized by SCLC cells, with higher efficiency of H446 OE-Exo. These observations suggest that exosomes in SCLC can facilitate the transfer of LYPLAL1-DT to other tumor cells.

### Exosomal LYPLAL1-DT enhances the malignant phenotype of SCLC cells

**Figure 2:**
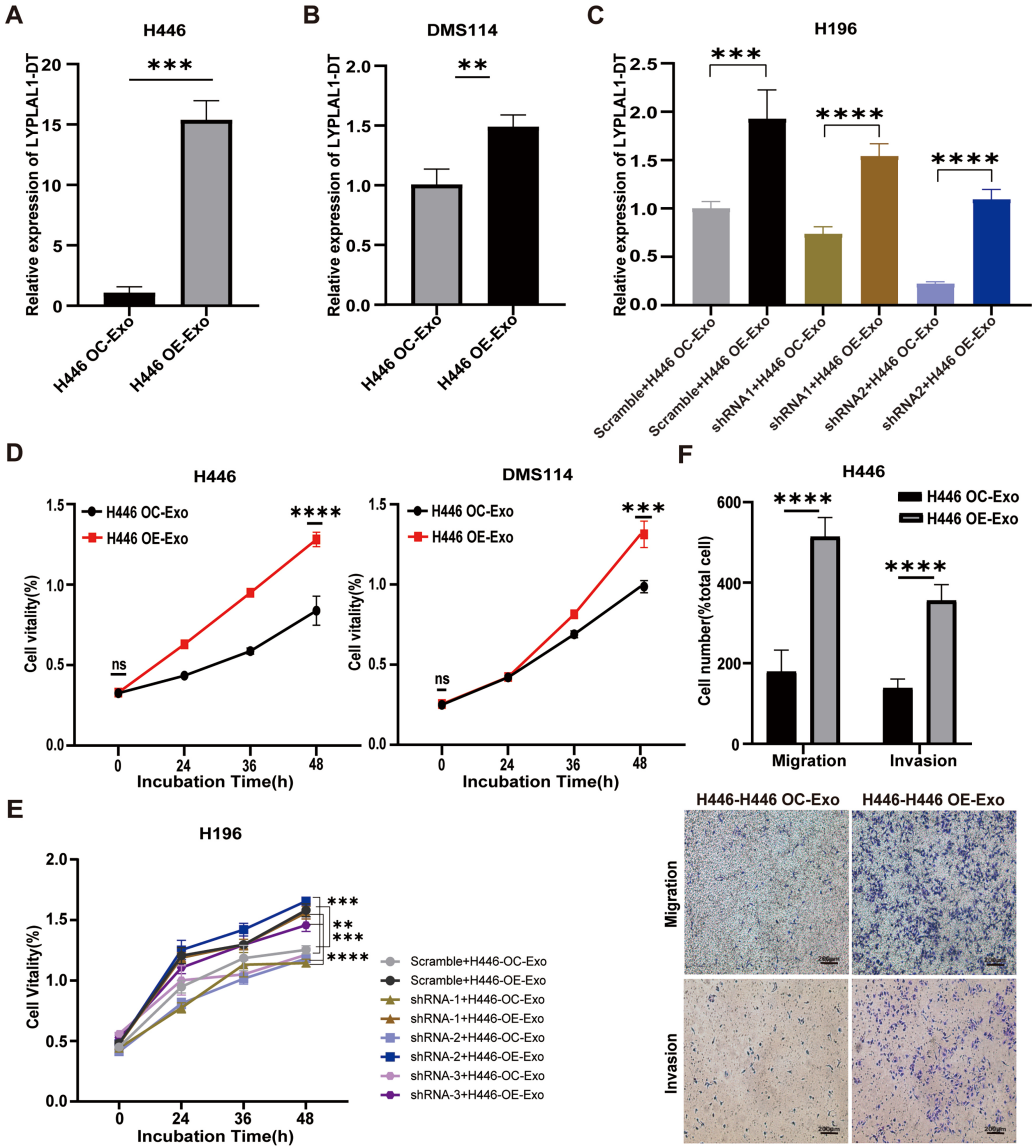
Exosomal LYPLAL1-DT promotes the malignant phenotype of SCLC cells. Various SCLC cell lines were treated with H446 OE-Exo or H446 OC-Exo. (A-C) The expression level of LYPLAL1-DT was significantly higher in the OE-Exo-treated group compared to the OC-Exo-treated group; (D and E) Cellular proliferation of H446 and DMS114 cells was significantly enhanced in the OE-Exo-treated group relative to their respective controls; (F) Cell invasion and migration were significantly increased in the OE-Exo-treated group (scale bar: 200 μm). Data are presented as mean ± SD. Differences between the two groups were analyzed by Student’s *t*-test, while comparisons among more than two groups were analyzed by one-way ANOVA. (^*^P < 0.05; ^**^P < 0.01; ^***^P < 0.001; ^****^P < 0.0001). LYPLAL1-DT: LYPLAL1 divergent transcript; SCLC: small cell lung cancer; OE-Exo: exosomes derived from LYPLAL1-DT-overexpressing cells; OC-Exo: exosomes derived from overexpression control cells; SD: standard deviation; ANOVA: analysis of variance.

Recently, exRNAs from tumor-derived EVs have been ascribed important functions in cancers. Tumor-derived RNA cargos mediate pro-tumorigenic effects by delivering specific regulatory molecules that facilitate oncogenic transformation and disease development^[37,38]^. To explore the functional role of exosomal LYPLAL1-DT in SCLC progression, we treated various SCLC cell lines, wild-type H446 cells (H446-WT), wild-type DMS114 cells (DMS114-WT) and LYPLAL1-DT knockdown H196 cell lines (referred to as shRNA-1, -2 and -3-H196 cells) with exosomes derived from H446-OE and H446-OC cells to investigate their effects on cell proliferation, migration, and invasion. After 24 h of exosome treatment, the expression of LYPLAL1-DT in SCLC cell lines was directly measured to assess whether exosomal LYPLAL1-DT was successfully delivered and internalized. The results demonstrated that the intracellular expression of LYPLAL1-DT was significantly higher in SCLC cells treated with H446 OE-Exo compared to those treated with control H446 OC-Exo [Figure 2A-C]. Correspondingly, the proliferation of H446 OE-Exo-treated various SCLC cells was significantly enhanced [Figure 2D and E]. A significant promotion of cell migration and invasion was observed in H446 cells following OE-Exo treatment compared with the control [Figure 2F]. Collectively, these data show a strong positive correlation between LYPLAL1-DT levels within SCLC cells and those packaged into secreted exosomes. It suggests that exosomal LYPLAL1-DT promotes the proliferation, migration, and invasion of SCLC cell lines, highlighting the critical role of exosomal LYPLAL1-DT in tumor progression.

### Exosomal LYPLAL1-DT promotes tumor angiogenesis of SCLC

**Figure 3:**
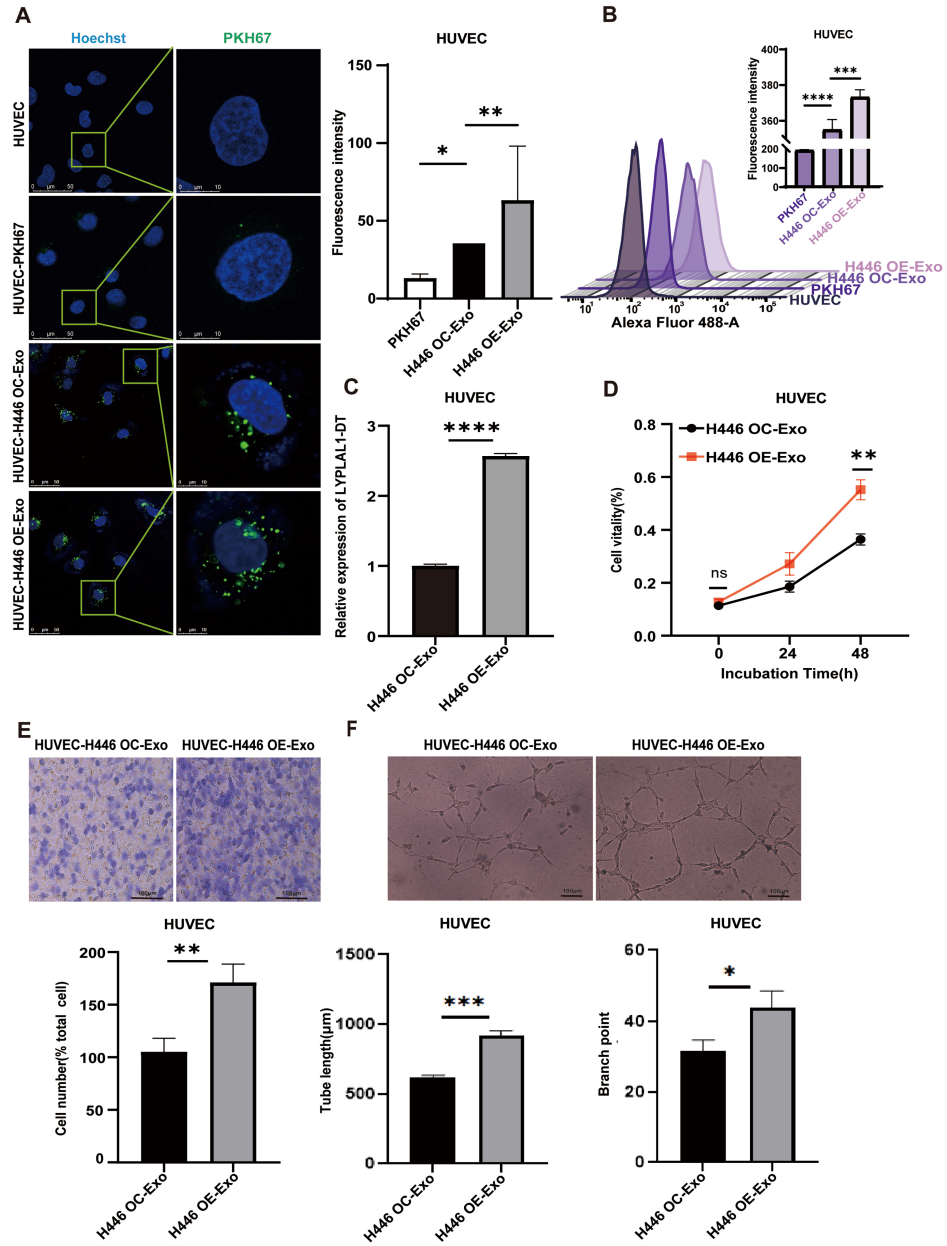
Effects of exosomal LYPLAL1-DT on angiogenesis of HUVEC. (A and B) H446-derived exosomes can be internalized by HUVEC (Scale bar: 50 and 10 μm); (C) The expression of LYPLAL1-DT in HUVECs was significantly higher following treatment with H446 OE-Exosomes compared to H446 OC-Exosomes; (D) The proliferation ability of HUVECs was significantly enhanced by H446 OE-Exosome treatment; (E) The migration ability of HUVECs was significantly increased by H446 OE-Exosome treatment; (F) The tube formation ability (tube length and branch point) of HUVECs was significantly promoted by H446 OE-Exosome treatment (scale bar: 100 μm). Data are presented as mean ± SD. Differences between the two groups were analyzed by Student’s *t*-test, while comparisons among more than two groups were analyzed by one-way ANOVA. (^*^P < 0.05; ^**^P < 0.01; ^***^P < 0.001; ^****^P < 0.0001). LYPLAL1-DT: LYPLAL1 divergent transcript; HUVEC: human umbilical vein endothelial cell; OE-Exo: exosomes from overexpressing cells; OC-Exo: exosomes from control cells; SD: standard deviation; ANOVA: analysis of variance; PKH67: PKH67 green fluorescent cell linker.

Tumors require a continuous supply of oxygen and nutrients for growth. To meet this demand, they stimulate the proliferation and migration of endothelial cells to form new blood vessels^[39,40]^. EV-RNA contributes to tumor expansion and dissemination by fulfilling a pro-angiogenic role, which is critical for these processes^[41,42]^. To investigate the impact of SCLC-derived exosomal LYPLAL1-DT on tumor angiogenesis, exosomes were isolated from H446-OE and control H446-OC cells, labeled with PKH67 (green), and added to the culture medium of HUVEC cells. Green fluorescence surrounding the nuclei of HUVEC cells under fluorescence microscope [Figure 3A] and a clear rightward peak shift in flow cytometry [Figure 3B] confirmed the successful uptake of H446 OE-Exo and H446 OC-Exo by HUVEC cells.

Subsequently, HUVEC cells were treated with the exosomes from either H446-OE or H446-OC cells for 24 h. qRT-PCR analysis revealed that exosomes derived from H446-OE cells elevated the expression level of LYPLAL1-DT in HUVEC cells more significantly than those from H446-OC cells [Figure 3C].

To evaluate the functional effects of tumor exosomes on endothelial cells, we assessed the proliferation and migration of HUVEC cells, which are essential for vascular network formation surrounding the tumor. The results demonstrated that HUVEC cells treated with exosomes from H446-OE cells exhibited significantly enhanced proliferation and migration compared to those from H446-OC cells [Figure 3D and E]. Furthermore, HUVEC cells produced significantly longer tube-like structures when exposed to conditioned medium from the H446-OE group than from the H446-OC group in a tube formation assay [Figure 3F]. These findings indicate that exosomal LYPLAL1-DT from SCLC cells significantly enhances endothelial cell angiogenesis, highlighting its pro-angiogenic potential in the TME.

### HUVEC-derived exosomal LYPLAL1-DT exerts a feedback influence on SCLC cells, enhancing their tumorigenic properties

**Figure 4:**
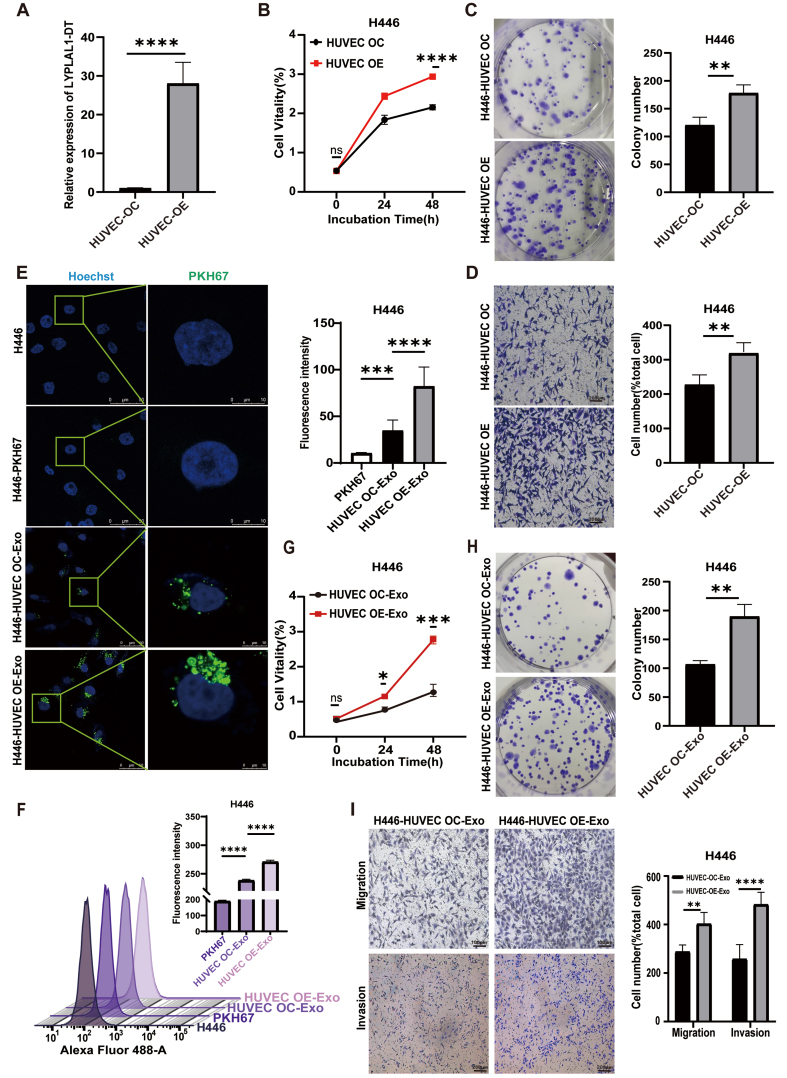
Enhanced proliferation, migration, and invasion of H446 cells mediated by HUVEC-derived exosomal LYPLAL1-DT. (A) The expression levels of LYPLAL1-DT were assessed in HUVECs with LYPLAL1-DT OE and OC; (B and C) When H446 cells were co-cultured with LYPLAL1-DT-OE-HUVECs, their proliferation capacity was significantly greater than that of cells co-cultured with control HUVECs; (D) The migration ability of H446 cells in the OE co-culture group was significantly greater than that in the OC group (scale bar: 200 μm); (E and F) PKH67-labeled exosomes derived from HUVECs were internalized by H446 cells (scale bars: 50 and 10 μm); (G and H) H446 cells treated with OE-Exo exhibited significantly enhanced proliferation compared to those treated with OC-Exo; (I) OE-Exo treatment significantly enhanced the migration and invasion of H446 cells compared to OC-Exo treatment (scale bar: 100 μm). Data are presented as mean ± SD. Differences between the two groups were analyzed by Student’s *t*-test, while comparisons among more than two groups were analyzed by one-way ANOVA. (^*^P < 0.05; ^**^P < 0.01; ^***^P < 0.001; ^****^P < 0.0001). HUVEC: Human umbilical vein endothelial cells; LYPLAL1-DT: LYPLAL1 divergent transcript; OE-Exo: exosomes from overexpressing cells; OC-Exo: exosomes from control cells; SD: standard deviation; ANOVA: analysis of variance; PKH67: PKH67 Green Fluorescent Cell Linker.

Emerging evidence suggests that the transfer of EV-RNAs constitutes a key mechanism in tumor-stromal crosstalk. This interplay, particularly between tumor and endothelial cells, thereby promotes tumor progression and metastatic dissemination^[43]^. To investigate whether the overexpression of LYPLAL1-DT in HUVECs could influence tumorigenesis, we first confirmed a significant increase in LYPLAL1-DT expression in the overexpressing cell lines [Figure 4A]. H446 cells were then co-cultured with either HUVEC-OE or HUVEC-OC using a transwell system for 24 h. We evaluated the proliferative and migratory capacities of H446 cells following the experimental treatment. Compared to the HUVEC-OC co-culture group, H446 cells co-cultured with HUVEC-OE cells exhibited significantly enhanced proliferation and migration [Figure 4B-D]. These findings indicate that LYPLAL1-DT overexpression in HUVECs can act on SCLC cells to promote their tumorigenic properties.

To explore whether exosomes mediate the observed effects of HUVEC-OE on H446 cells, exosomes were extracted from the conditioned medium of HUVEC-OE and HUVEC-OC cells. Uptake experiments confirmed that exosomes derived from both HUVEC-OE and HUVEC-OC cells were successfully internalized by SCLC cells [Figure 4E and F]. To further investigate the role of exosomal LYPLAL1-DT from EC, SCLC cells were treated with exosomes from either HUVEC-OE (HUVEC OE-Exo) or HUVEC-OC (HUVEC OC-Exo) cells. As expected, compared to the HUVEC OC-Exo group, SCLC cells treated with HUVEC OE-Exo exhibited significantly increased proliferation [Figure 4G and H] and enhanced migration and invasion capabilities [Figure 4I].

These results demonstrate that LYPLAL1-DT derived from HUVEC exosomes significantly promotes the proliferation, migration, and invasion of SCLC cells, suggesting that EC could reversely contribute to SCLC tumor formation through exosomal LYPLAL1-DT.

### Exosomal LYPLAL1-DT mediates tumorigenesis and angiogenesis through miR-204-5p/PFN2, miR-204-5p/BCL2 and miR-204-5p/SIRT1 axes

In our previous study, we have identified that LYPLAL1-DT facilitates SCLC tumorigenesis and chemoresistance through a ceRNA mechanism involving the miR-204-5p/PFN2 and miR-204-5p/BCL2 axes^[15,16,20]^. Furthermore, we have elucidated that LYPLAL1-DT promotes HUVEC proliferation and modulates systemic inflammation via miR-204-5p/SIRT1 axis, conferring a protective effect on endothelial cells^[14]^. In the present study, we investigated the molecular mechanisms underlying the bidirectional crosstalk between SCLC and endothelial cells involving exRNA LYPLAL1-DT.

**Figure 5:**
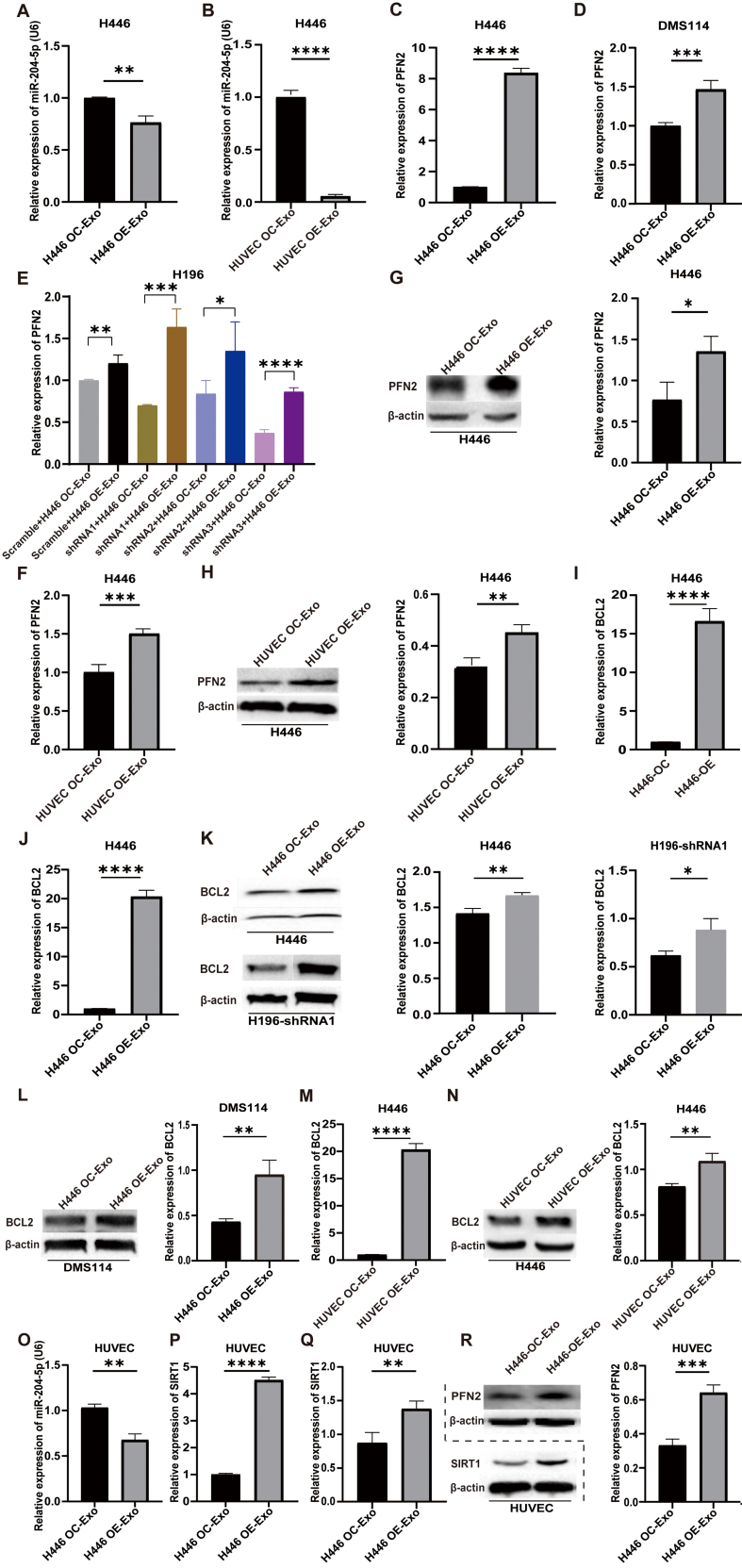
MiR-204-5p/PFN2, miR-204-5p/BCL2 and miR-204-5p/SIRT1 axes were involved in exosomal LYPLAL1-DT mediates tumorigenesis and angiogenesis. (A and B) MiR-204-5p levels in H446 cells were measured by qPCR following treatment with exosomes from LYPLAL1-DT-overexpressing H446 cells (OE-Exo) (A) or HUVEC cells (OE-Exo) (B); (C and D) PFN2 mRNA expression was determined by qPCR in H446 (C) and DMS114 (D) cells after treatment with exosomes from LYPLAL1-DT-overexpressing-H446 cells (OE-Exo) or control cells (OC-Exo); (E) PFN2 expression was analyzed by qPCR in LYPLAL1-DT-knockdown H196 cells treated with exosomes from LYPLAL1-DT-overexpressing-H446 cells (OE-Exo) or control cells (OC-Exo); (F) PFN2 mRNA levels in H446 cells exposed to exosomes from LYPLAL1-DT-overexpressing HUVEC cells (OE-Exo) or control cells (OC-Exo); (G and H) PFN2 protein levels were detected by Western blotting in H446 cells treated with exosomes from LYPLAL1-DT-overexpressing H446 cells (OE-Exo) (G) or HUVEC cells (OE-Exo) (H); (I) The expression level of BCL2 was measured in H446 cells when co-cultured with LYPLAL1-DT-OC- or OE- H446 cells; (J-L) The expression level of BCL2 in H446, H196 and DMS114 cells when treated with exosomes from LYPLAL1-DT-overexpressing-H446 or control cells (OC-Exo); (M and N) The expression level of BCL2 in H446 cells treated with exosomes from LYPLAL1-DT-overexpressing-HUVEC cells (OE-Exo) or control cells (OC-Exo) was measured by qRT-PCR and Western blotting; (O-R) HUVEC cells treated with exosomes from LYPLAL1-DT-overexpressing-H446 cells (OE-Exo) or control cells (OC-Exo). The expression levels of miR-204-5p (O), SIRT1 (P and Q) and PFN2 (R) were tested by qPCR and Western blot. Data are presented as mean ± SD. Differences between the two groups were analyzed by Student’s *t*-test, while comparisons among more than two groups were analyzed by one-way ANOVA. (^*^P < 0.05; ^**^P < 0.01; ^***^P < 0.001; ^****^P < 0.0001). . MiR-204-5p: MicroRNA-204-5p; PFN2: profilin 2; BCL2: B-cell lymphoma 2; SIRT1: sirtuin 1; LYPLAL1-DT: LYPLAL1 divergent transcript; OE-Exo: exosomes from overexpressing cells; OC-Exo: exosomes from control cells; HUVEC: human umbilical vein endothelial cells; qPCR: quantitative polymerase chain reaction; SD: standard deviation; ANOVA: analysis of variance.

Wild-type SCLC cell lines were treated with exosomes derived from H446-OE or HUVEC-OE cells, respectively. Total RNA and protein were extracted for qPCR and Western blot analyses. The results demonstrated that treatment of exosomes from H446-OE and HUVEC-OE cells markedly decreased the expression level of miR-204-5p [Figure 5A and B] and increased PFN2 expression in three wild-type SCLC cells (H446, H196 and DMS114 cells), compared to controls treated with H446-OC or HUVEC-OC exosomes by qRT-PCR [Figure 5C-F] and Western blot [Figure 5G and H]. A similar expression change was also observed in BCL2, a key regulator of apoptosis and autophagy [Figure 5I-N].

In HUVECs treated with exosomes from H446-OE cells, miR-204-5p levels were also notably reduced [Figure 5O]. In contrast, PFN2 and SIRT1 levels were significantly elevated [Figure 5P-R].

The results in different SCLC cell lines all suggest that exosomal LYPLAL1-DT promotes SCLC malignant phenotypes through miR-204-5p/PFN2 and miR-204-5p/BCL2 axes. In endothelial cells, exosomal LYPLAL1-DT enhances angiogenesis by modulating miR-204-5p, PFN2, and SIRT1 levels, underscoring its critical role in tumor progression and vascular remodeling.

## DISCUSSION

In the present study, we not only investigated the effect of exRNA LYPLAL1-DT on SCLC on different subtypes of SCLC cells but also examined the intercellular communication between SCLC and endothelial cells established through exRNA LYPLAL1-DT. This study highlights its critical role in the progression of SCLC, and its involvement in both tumorigenesis and angiogenesis. Our findings provide a comprehensive understanding of the molecular mechanisms through which exosomal LYPLAL1-DT facilitates crosstalk between tumor cells and endothelial cells, promoting malignant phenotypes and vascular remodeling in SCLC.

EVs act as critical mediators in tumor-stroma crosstalk, orchestrating the cellular interactions that support cancer progression and metastasis. Notably, cancer cells release significantly more EVs than non-malignant cells, driven by hyperactive oncogenic signaling pathways and deregulation of membrane fusion machinery^[44-46]^. As a key subclass of EVs, exosomes function as critical signaling hubs that integrate external stimuli and transduce them into phenotypic changes within tumor cells. The RNA molecules contained within exosomes not only provide insight into tumor status but also act as diagnostic and prognostic markers^[47]^. In the present study, elevated levels of exosomal LYPLAL1-DT were detected in patient serum, suggesting active secretion by SCLC cells into the TME. Moreover, our data demonstrated that SCLC cells efficiently internalize exosomal LYPLAL1-DT, potentially amplifying its functional effects within the tumor milieu. Our study further revealed that exosomal LYPLAL1-DT significantly enhances the malignant phenotype of SCLC cells, driving their proliferation, migration, and invasion. These findings align with the growing body of evidence that exosomes act as carriers of oncogenic signals, facilitating the horizontal transfer of functional molecules^[48]^. PFN2, an actin cytoskeleton regulator, plays a crucial role in cell motility, with its function varying across different tumor types. Mouneimne *et al*. proposed that PFN2-mediated actin polymerization enhances actin bundling, thereby suppressing breast cancer cell invasion^[49]^. In contrast, Toyota *et al*. examined the expression levels of PFN2 in multiple metastatic cells and found its upregulation related to lung and colorectal cancer cell motility, adhesion, and migration^[50]^. In our previous study, we demonstrated that PFN2 upregulation promotes tumorigenesis and invasion in SCLC *in vitro* and *in vivo*^[15,20]^. Accordingly, exosomal LYPLAL1-DT may induce cytoskeletal remodeling and enhance cellular motility by increasing PFN2 levels in recipient cells. This provides a plausible explanation for the observed increase in SCLC aggressiveness.

BCL2, an anti-apoptotic protein, plays a crucial role in the regulation of cell survival, and its expression is often dysregulated in various cancers, contributing to tumorigenesis and the resistance of cancer cells to cell death signals^[21,22]^. We previously demonstrated that LYPLAL1-DT acts as a molecular sponge for miR-204-5p, leading to the upregulation of BCL2 and consequent inhibition of chemotherapy-induced apoptosis in SCLC. Based on this finding, we evaluated a combination therapy targeting BCL2, which significantly suppressed tumor growth in various xenograft models, thereby validating its therapeutic potential^[16]^. In this study, we observed that the expression level of BCL2 was significantly upregulated when SCLC cells internalized exosomes. That indicates the increased cell survival, enhanced proliferation and resistance to chemotherapy of tumor cells. This is consistent with the biological characteristics of SCLC.

Angiogenesis is a prerequisite for tumor expansion and metastasis. Endothelial cells can be stimulated to sprout and initiate angiogenesis by pro-angiogenesis factors^[51]^. Recent advances have deconvoluted the complexity of the angiogenic program during tumor growth at single-cell resolution across 31 cancer types, providing a molecular landscape of tumor angiogenesis^[52]^. We previously identified a protective role for LYPLAL1-DT in endothelial cells, which it exerts by acting as a ceRNA for miR-204-5p to elevate SIRT1 expression^[14]^. In the present study, HUVEC cells exhibited significantly enhanced proliferation and migration ability when treated with the exosomes from H446-OE cells. This was accompanied by increased exosome internalization and upregulation of intracellular LYPLAL1-DT levels. Intriguingly, our study also revealed a reciprocal relationship: HUVEC-derived exosomal LYPLAL1-DT enhances the tumorigenic properties of SCLC cells. This bidirectional crosstalk between tumor cells and endothelial cells underscores the complexity of the TME and highlights exosomal LYPLAL1-DT as a central mediator in this interplay.

**Figure 6:**
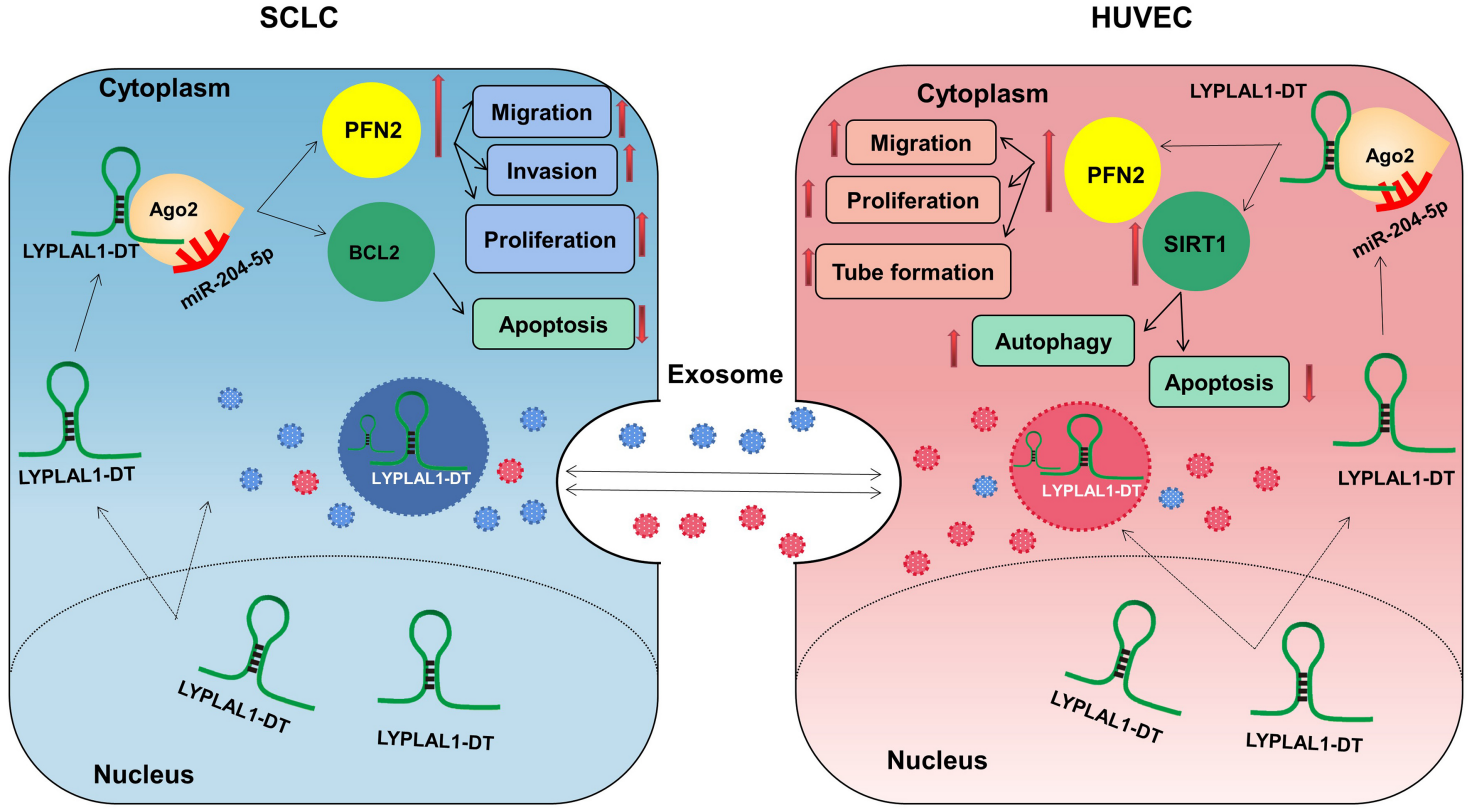
Schematic diagram of exosomal LYPLAL1-DT-mediated crosstalk between SCLC and HUVE cells (Original, Created with Adobe Illustrator 2022). LYPLAL1-DT: LYPLAL1 divergent transcript; SCLC: small cell lung cancer; HUVEC: human umbilical vein endothelial cells; miR-204-5p: microRNA-204-5p; PFN2: profilin 2; BCL2: B-cell lymphoma 2; SIRT1: sirtuin 1; Ago2: argonaute RISC catalytic component 2.

LncRNAs employ several fundamental mechanisms to regulate gene expression, including acting as signals, decoys, guides, and scaffolds^[53,54]^. They can function as ceRNAs, sequencing or inhibiting miRNA activity by binding to target mRNA. Our previous studies have identified the LYPLAL1-DT/miR-204-5p/PFN2 and LYPLAL1-DT/miR-204-5p/BCL2 axes in SCLC cells^[15,16]^, and LYPLAL1-DT/miR-204-5p/SIRT1 axis in HUVECs^[14]^ by RNA immunoprecipitation (RIP), knockdown or overexpression, and rescue experiments. On this basis, we verified that exosomes derived from HUVECs and tumor cells containing LYPLAL1-DT were efficiently internalized by SCLC cells, leading to decreased miR-204-5p levels and increased PFN2 and BCL2 expression in the recipient tumor cells. These data further clarify the regulatory axes we previously demonstrated. Similarly, in endothelial cells, tumor cell-derived exosomal LYPLAL1-DT downregulated miR-204-5p, resulting in elevated levels of both PFN2 and SIRT1. SIRT1 plays a well-established role in promoting endothelial cell survival and angiogenesis, further supporting its involvement in exosomal LYPLAL1-DT-mediated vascular remodeling. These findings not only provide new insights into the high levels of LYPLAL1-DT observed in the cytoplasm, but also complete the loop of LYPLAL1-DT-mediated crosstalk between SCLC and endothelial cells [Figure 6].

Our study has certain limitations that should be considered. The relatively small sample size for the serum validation of exosomal LYPLAL1-DT may limit the statistical power. Future studies with larger, multi-center cohorts are necessary to confirm the diagnostic or prognostic value of LYPLAL1-DT. While we have identified the key roles of LYPLAL1-DT in the exosome, the functions of other components have yet to be elucidated. It remains unclear whether PFN2, BCL2 or SIRT1 are simultaneously present in exosomes, or if the observed effects are associated with the uptake of these factors. Addressing this gap will enhance our understanding of the TME and potentially reveal additional therapeutic targets. Furthermore, although we demonstrated the involvement of exosomal LYPLAL1-DT in SCLC progression and angiogenesis, the specific mechanisms related to the cell cycle, tumor development, and angiogenesis require further investigation. Lastly, although we have identified ELAVL4 as an upstream regulator of LYPLAL1-DT^[15]^, other regulators of exosomal LYPLAL1-DT biogenesis and secretion should be unraveled. Elucidating these mechanisms could provide deeper insights into its role in SCLC pathogenesis.

## DECLARATIONS

### Availability of data and materials

All relevant data are included within the paper. The data that support the findings of this study are available from the corresponding author upon reasonable request.

### Copyright

© The Author(s) 2025.
